# Municipal governments’ perspectives on forest ownership: Insights from Czechia

**DOI:** 10.1007/s13280-025-02231-8

**Published:** 2025-08-09

**Authors:** Vojtěch Kotecký, Vojtěch Kosour, Vojtěch Čemus, Rozálie Stejskalová, Petr Pavelčík

**Affiliations:** 1 Environment Centre, Charles University in Prague, José Martího 407/2, 162 00 Prague 6, Czechia; 2 Faculty of Humanities, Charles University in Prague, Pátkova 2137/5, 182 00 Prague 8, Czechia; 3 CI2, Oldřichova 517/33, 128 00 Prague 2, Czechia

**Keywords:** Community forestry, European forestry, Landowner objectives, Multifunctionality, Municipal forests, Recreational woodland

## Abstract

Municipal ownership accounts for a large proportion of the forest area in some European countries. Research on local government forestry objectives has so far focused on green spaces in and near built-up areas, and on countries with relatively low shares (< 5%) of municipal forest land. We performed a nationwide quantitative survey of preferences of municipalities in Czechia, a country where they own a major share (17%) of forest land. Here we show that municipalities prioritise recreation and certain environmental objectives over income generation, even in non-urban contexts. However, local governments also emphasise continuity, potentially—albeit inadvertently—prolonging a pre-existing focus on timber production. We found surprisingly little variation in the importance given to recreation across various municipal characteristics.

**Supplementary Information:**

The online version contains supplementary material available at 10.1007/s13280-025-02231-8.

## Introduction

Forestry scholars and policymakers in Europe increasingly emphasise multifunctional management (European Academies Science Advisory Council 2017; European Commission 2021). But practical implementation depends largely on the preferences and choices of forest owners. They make the ultimate decisions on forest priorities and management, albeit within a regulatory framework (Nichiforel et al. 2018).

European forest ownership is a patchwork of state, private, community, church and municipal property (Pulla et al. 2013; Živojinović et al. 2015; FAO and UNECE 2019). Municipal forests, institutionally owned by local governments, are a longstanding, specifically European tradition. Institutional public ownership usually distinguishes municipal property from commons—land collectively shared by a group of private individuals, typically members of a historical local community—which are widespread in the global South and present but rare in Europe (FAO and UNECE 2019; Lawrence et al. 2021).

The European Federation of Forest-Owning Communities estimates that local authorities own around 12.5% (approximately 20 million hectares) of forest land in the European Union (FECOF 2014). The percentage ranges from 10 to 36% in several countries including Luxembourg, Switzerland, Germany, Czechia, France, the Netherlands, Bulgaria and the region of Wallonia, with more than 50% in Albania and the German state of Rhineland-Palatinate. Although municipal ownership in Central and Eastern Europe was temporarily affected by communist rule, the percentage does not appear to follow a substantial east–west divide. The current distribution reflects deeper histories as well as outcomes of two more recent developments: the diverse trajectories of land ownership in post-World War 2 democratic Europe, and the heterogeneity of restitution policies in post-communist countries in the 1990s.

Municipalities inevitably face conflicting forest priorities and the trade-offs they entail. European city governments have negotiated contradictory uses of municipal forest land since the Middle Ages (Radkau 2012). Municipal forests are usually considered a specific form of public land and as such might be expected to contribute to public objectives. The EU Forest Strategy argues that “[i]n publicly owned forests it is only reasonable…to strengthen forest protection and restoration efforts to achieve the commonly agreed increased EU climate and biodiversity ambition” (European Commission 2021: 16). However, municipal properties occupy an uncertain position shaped by two contrasting perspectives of public ownership: the presumable embodiment of non-market services, and a private-like distance from central government policy priorities. This equivocality leads to some confusion, with some national statistics including municipal property under public forests and others considering it private property (Živojinović et al. 2015).

A substantial body of research has investigated evolving values, motivations, attitudes and goals of forest owners, with a strong focus on small private landowners. It suggests that, alongside material trade-offs between timber production and other objectives, there is a genuine heterogeneity of views among private owners, shaped by ongoing demographic and social changes (Weiss et al. 2019). A number of traditional farm-based owners remain focused on production and are embedded in a rural identity. Meanwhile, younger, urban, absentee, educated and female individuals or families, often professionally and culturally disconnected from rural land-based lifestyles, increasingly emphasise a shift from an exclusive focus on raw material output to objectives revolving around a mix of market and non-market uses. This aligns with a broader shift in societal preferences towards multifunctional management and non-market forest values (Tarrant and Cordell 2002; Bengston et al. 2004; Rametsteiner et al. 2009; Krejčí et al. 2019). Researchers in European countries have proposed a number of formal typologies of private owners based on their attitudes and priorities (for overviews see Ní Dhubháin et al. 2007 and Ficko et al. 2019). Evolving values impact silvicultural choices (Pynnönen et al. 2018) and, consequently, harvesting behaviour (Kuuluvainen et al. 2014; Heinonen et al. 2020) or local biodiversity (Koskela and Karppinen 2021; Hansen et al. 2023). It is only natural that this research focuses on small private owners. As they control 56% of forest area in the 28 European countries with available data (FAO and UNECE 2019), their preferences have a major impact on the continent’s forests.

But a similar insight is lacking for municipal forests. Research suggests that European municipalities’ objectives gradually shift towards multifunctional use of forest land (Mattila et al. 2013). Nielsen et al. (2013) found that Danish local governments prioritised recreation and nature conservation more often than timber production in their forest management plans. In a Swedish case study, municipal owners also emphasised multiple—market and non-market—functions of their forest land (Richnau et al. 2013). However, to date, research has focused on Nordic countries, which tend to have comparatively low levels (< 5%) of municipal forest ownership. They are not necessarily representative of jurisdictions where municipalities are a major segment of forest owners. Large private forest owners are known to prioritise timber production over non-market functions more than small landholders do (Eggers et al. 2014; Blanco et al. 2015; Tiebel et al. 2022).

Much research on local government forest ownership in Europe has emphasised urban and peri-urban spaces with a focus on recreational services, largely overlapping with the field of urban forestry research (Konijnendijk et al. 2006). The focus on the overlap between municipal ownership and urban forestry is understandable, but too narrow. Forests are an important environmental resource in European urban areas (Hunter 2001; Hansen-Møller and Oustrup 2004; Konijnendijk et al. 2005), and local governments own the majority of forests in some large cities. In Denmark, 83% of the surveyed municipal woodlands were located within or on the outskirts of urban settlements (Nielsen et al. 2013). About 30% of Finnish forest visitors use municipally owned land, even though this only accounts for 1.6% of the country’s forest area (Mattila et al. 2013).

Nevertheless, municipal forests in many European countries are a much broader phenomenon. While urban woodlands are, by definition, situated in and near built-up sites, many municipal forests are production forests outside urban areas. Indeed, many of the owners are rural municipalities. Even in the Nordic countries with comparatively low levels of municipal ownership, the total area of municipal forest is almost twice that of urban woodland (narrowly defined, i.e. those with uncultivated ground vegetation), which in turn includes substantial non-municipal properties (Gundersen et al. 2005; Nielsen et al. 2013; FAO and UNECE 2019).

A better understanding of the priorities of forest owning municipalities in Europe is important to gain a broader perspective. Here we investigate the objectives and preferences of municipalities in Czechia. Among the European countries with significant municipal forest ownership, Czechia is an interesting case from research perspective. The country has the most decentralised (per mean population of municipality) local governments in the EU (Mackie and Thijs 2021). As small villages are governed independently, the resulting broad range of municipal sizes provides an opportunity to investigate preferences across unusually wide gradients of town sizes, budgets and socio-demographic variables.

We focus on three key research questions: (i) what are the priorities of forest owning municipalities? (ii) Are the priorities linked to the material needs of municipalities (importance of forest income in local government budgets and recreational needs)? (iii) Are the priorities related to the character of the municipality (population size, rural and peripheral character)?

## Materials and methods

### Study population

Czechia is a medium-sized (area 78,871 km^2^, population 10.5 million), moderately affluent (GDP PPP US$ 53 080 per capita) European Union economy with 34.2% forest cover. Forestry and logging contribute 0.4% to GDP (Eurostat 2022). Forests are dominated by Norway spruce *Picea abies* (46.0%), Scots pine *Pinus sylvestris* (16.0%), European beech *Fagus sylvatica* (9.8%) and oaks *Quercus* sp. (7.9%) (Ministry of Agriculture 2024). The country’s spruce forest resources and timber markets have been significantly affected by a major bark beetle outbreak after 2017 (Hlásny et al. 2021). The forest land nationalised since the communist takeover in 1948 was gradually returned to former private, municipal and church owners after 1989, but the majority (53.7%) of forests remain in central government ownership (Ministry of Agriculture 2024).

The population of the 6254 Czech municipalities ranges from 16 to 1 357 326, and only 2% of municipalities have more than 10 000 inhabitants. Many of the municipalities own 17.2% of the country’s forests (449 000 ha), a share only slightly lower than that of private owners (23.7%) (Ministry of Agriculture 2024). Most of them are small towns and villages: while 22.4% of the Czech population live in cities with > 100 000 inhabitants, they own only 4.8% of municipal forests.

Municipal forest ownership in Czechia has emerged over the centuries through purchases, royal donations, afforestation and transformation of commons, as well as two waves of land reform in 1919 and 1945. The continuity of ownership was interrupted for several decades after 1948. Since the restitution process ended around 2005, the municipal share of forest ownership has remained almost unchanged.

We conducted a nationwide online survey (Gideon 2012) of a random sample (*N* = 520) of municipalities that are forest owners. Following consultations with practitioners who provide forest management services to municipalities, we limited the population to municipalities with a valid forest management plan (*N* = 1821), excluding those with small properties that tend to be materially insignificant in their governance choices. A database operated by the government’s Forest Management Institute was used to identify the relevant municipalities. A questionnaire (“Data collection” section) was e-mailed to the general contact addresses provided on the local government websites, along with a brief introductory message and a request to forward it to an appropriate person responsible for forestry decision-making in the municipality. Municipalities that failed to respond were sent a reminder after three weeks.

### Data collection

For the online survey, we designed a self-administered, structured questionnaire with 27 items to measure the respondents’ views and preferences (Table 1), using two principal strategies. First, we inquired directly about municipalities’ ownership objectives and current management goals. Secondly, we gauged respondents’ priorities using a series of items similar to willingness-to-pay questions to explore their willingness to forgo some timber income in favour of other priorities, and asked about their motivations for potential forest certification as a proxy measure for practical action. In addition, we included four binary items testing willingness to enter FSC and PEFC certification schemes (considering; potentially useful but not considering; useless for the municipality; not aware of the certification), as well as five factual questions (the position of the respondent within the local government; the area of municipal forest owned; the percentage of timber sales in the local government income on an interval scale; and participation in the two certification schemes as a measure of proactive approach to forest management). We pre-tested the questionnaire in semi-structured interviews with 10 municipalities, and in a preliminary survey of a limited sample of municipalities.

**Table 1:** Questionnaire items used to gauge municipalities’ views and priorities of forest ownership

Theme	Items	Values
Ownership objectives		5-point scales for each item (Strongly agree; Agree; Disagree; Strongly disagree; Do not know)
Indexed as recreational	Recreation	
	Non-wood forest products	
	Sport	
Indexed as economic	Timber sales	
	Fuelwood provision	
Indexed as environmental	Water resources	
	Noise protection and/or microclimate	
	Flood protection	
Others	Continued tradition of forest ownership	
	School education	
	Game and hunting	
	Incoming tourism	
	Carbon sequestration	
Management goals	Management goals	2 preferred options: Environmental; Social; Economic; Cultural; Political decision; Continuation of pre-existing practice
Willingness to forgo some timber income for other priorities	Water resources	0%, 10%, 30%, 50% and 100% of the municipal income
	Biodiversity	
	Flood protection	
	Game and hunting	
	Recreation	
Potential motivation to enter a certification scheme	Higher timber price	5-point scales for each item (Strongly agree; Agree; Disagree; Strongly disagree; Do not know)
	Higher demand	
	Nature and biodiversity	
	Local participation in forest management	
	Better perception of forest management	
	Political choice	
	Prevention of diebacks	
	Government subsidies	

We extracted data on local population, businesses and land use directly from the Czech Statistical Office’s database of municipality profiles (Czech Statistical Office 2022), and calculated several additional indicators (“Data analysis” section and Table 4) from the data.

### Data analysis

We aggregated the ownership objective items into three indices for (i) economic objectives (timber sales, fuelwood provision), (ii) environmental objectives (water resources, noise protection and/or microclimate, flood protection) and (iii) recreational objectives (recreation, non-wood forest products, sport). We performed the data analyses in Minitab 17 software. Cronbach’s alpha was used to calculate the reliability of the scores of the relevant items. The values for all the ownership objective items ranged from 0.70 to 0.77, while the values for all the willingness to forego timber income items were around 0.95. These results suggest reasonable—and in the latter case, very high—rates of reliability, as 0.70 is often considered a cut-off value (Christmann and Aelst 2006).

We aggregated the items measuring willingness to forego timber income into two indices for (i) environmental priorities (biodiversity, water supply, flood protection) and (ii) recreational priorities (recreation, hunting). Each index was calculated as the mean of the respondent’s values for the constituent items. The reliability of these indices was again tested using Cronbach’s alpha, with values ranging from 0.86 to 0.93.

We performed an ordinal logistic regression to examine the possible relationships between ownership objectives with > 30% of strongly agree responses as well as the three ownership objectives indices and five descriptive variables: (i) two descriptive variables of municipal needs: the share of timber sales in the local government income (questionnaire item) and forest cover (%) as a proxy for recreational needs; and (ii) three descriptive variables of municipal character: population size (2022), population trend between 2001 and 2021 (%) and the share (%) of rural sectors (agriculture, forestry and fisheries) in the businesses registered in the municipality (2022). Similarly, an ordinal logistic regression was applied to test the relationship between each of the two indices of willingness to forego income for other priorities and the same five descriptive variables listed in (i) and (ii) above. We also used an ordinal logistic regression model to determine whether there is an interaction between the six descriptive variables of municipalities, the objectives of forest ownership and the position of the person who responded to the questionnaire (elected representative, forest manager, municipal staff, other).

## Results

We received 144 responses (28% response rate) to the questionnaire. The responding municipalities were located in all of the country’s administrative regions (*kraj*) except for the city of Prague. Their population and forest cover ranged from 73 to 279 791 residents (median: 1025) and from 1.0% to 80.3% (median: 37.1%), respectively. The self-reported area of municipal forest property ranged from 2.5 to 3320.0 (median: 100.0) ha. In 38.9% of the responding local governments, the share of timber sales was less than 1% of the income, and in another 29.9%, it was 1–5% (> 5% share in 21.5% of municipalities; 9.7% of respondents did not answer this item). Elected representatives completed 52.0% of the responses, forest managers 29.2% and municipal staff 18.8%. Small minorities of municipalities are certified under FSC (3.5%) and PEFC (18.1%) schemes.

### Municipal priorities

**Fig. 1:**
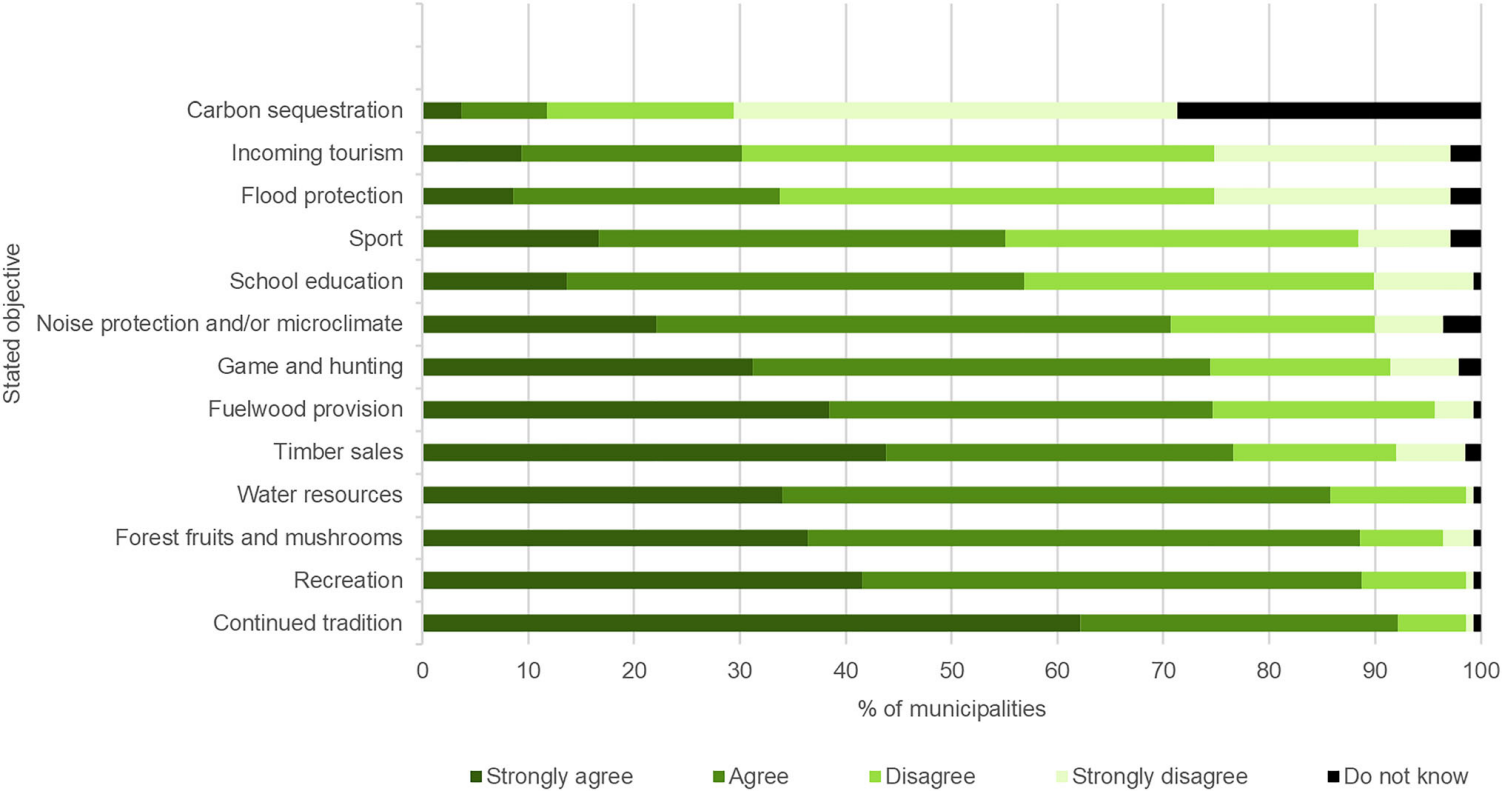
Stated objectives of municipalities’ forest ownership (*N* = 144), share of those giving a response

When asked about the objectives of forest ownership (Fig. 1), continued tradition of forest ownership was the most frequently selected option (92.1% of positive responses). Respondents also tended to select recreation (88.8%), non-wood forest products (88.6%) and water resources (85.8%) above timber sales (76.6%). Providing fuelwood to local citizens (74.6%) was considered almost as important as timber sales, but with a lower proportion of strongly positive responses. However, small minorities prioritised timber sales but no environmental or recreational objectives (4.4% each). Similarly, when asked to identify the two most important goals of the current forest management practice, more respondents gave priority to the environment than to the municipal economy or the continuation of pre-existing practices (Table 2).

**Table 2:** Most important goals of current forest management practice, by proportion of municipalities prioritising them. Respondents (*N* = 144) were asked to select the two most important goals. ^a^We received only 224 valid responses: 58 municipalities selected only one goal and six gave an invalid response (they usually selected more than two goals)

Goal	Share of respondents (%)^a^
Environmental	68.1
Economic	42.0
Continuation of a pre-existing practice	27.5
Political decision	8.0
Cultural	7.2
Social	5.1
Do not know	4.3

**Fig. 2:**
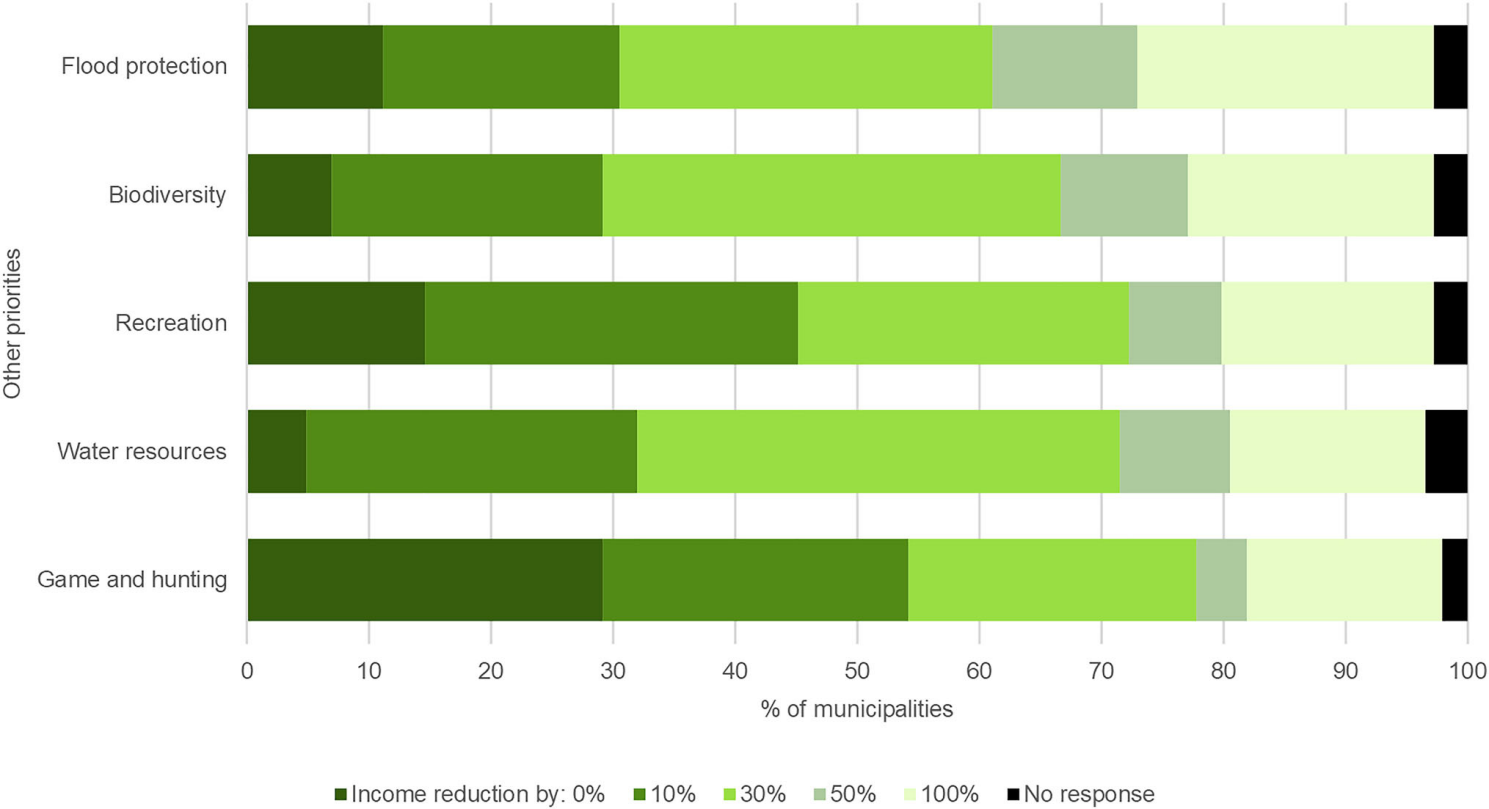
Municipalities’ willingness to forgo timber income for other priorities (*N* = 144), share of municipalities by the fraction of income

On average, municipalities were willing to forgo 35.0% of their timber income for another priority (Fig. 2). Willingness is almost unanimous; only 5.6% of responding municipalities were not ready to forgo timber income for any non-market priority. Willingness appeared to be similar across non-market priorities (Fig. 2), with the lowest responses in game and hunting (29.2% of municipalities were not willing to forgo any timber income for this priority).

**Fig. 3:**
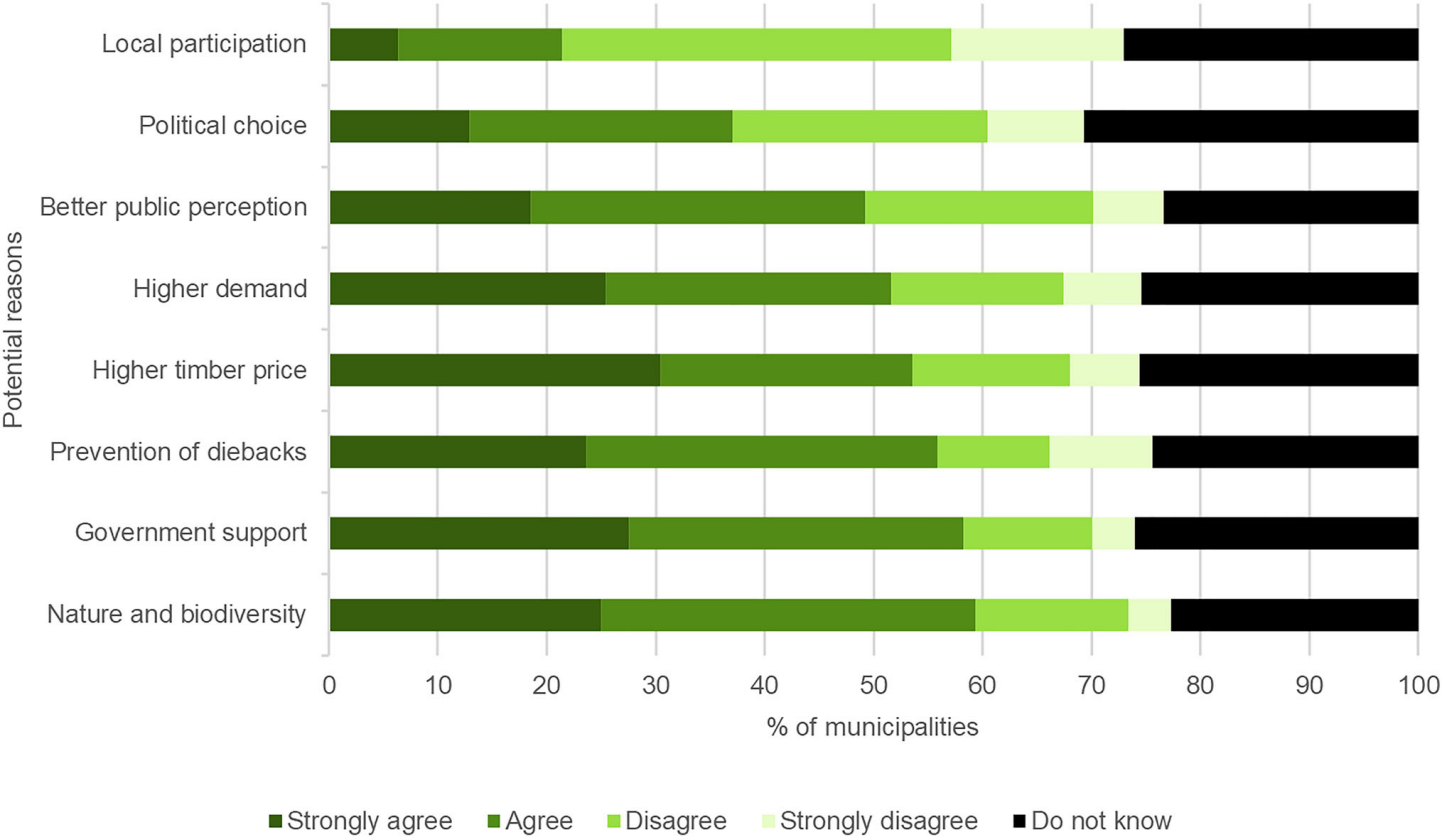
Potential reasons for forest certification (FSC or PEFC), (*N* = 144), share of those giving a response

Municipalities tend not to consider certification and have neither a positive nor a negative view of it: for a clear majority forest certification is not a relevant issue, with 76.4% of municipalities reporting no opinion on any of the certification schemes (other than their own non-participation). When prompted, respondents selected feasible motivations for certification (Fig. 3). However, given the generally passive attitude towards certification, these proclaimed motivations may have limited predictive power.

### Variation by municipal material needs

**Fig. 4:**
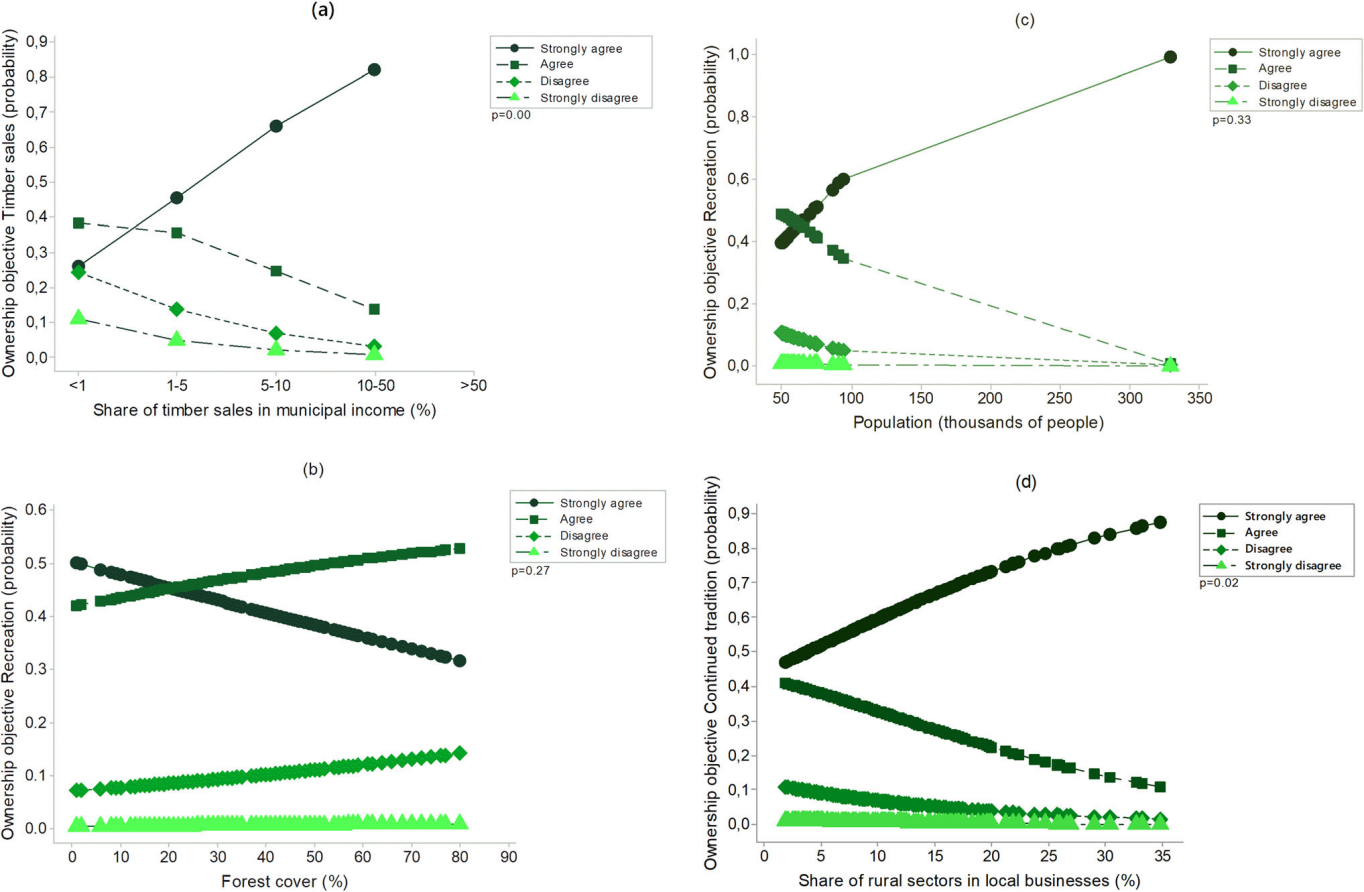
Ordinal logistic regression results for the relationships between **a** share of timber in the municipal income (%) and ownership objective timber sales, **b** forest cover (%) and ownership objective recreation, **c** population size and ownership objective recreation and **d** share of rural sectors in local businesses (%) and ownership objective continued tradition of forest ownership. Details of the ownership objective variables are given in Table 1

The share of timber sales in municipal income predicts timber sales, fuelwood provision and continued tradition of forest ownership as objectives of municipal forest ownership (Fig. 4a, Fig. S1 and Table 3). However, there is no significant relationship between the share of timber sales in the municipal income and the environmental objectives, nor with the willingness to forgo timber income for other priorities (Table 3).

**Table 3:** Summary of ordinal logistic regression results for relationships between ownership objectives and the importance of timber for municipal budget

		1–5% vs (0–1% and 5–10% and 10–50%)			5–10% vs (0–1% and 1–5% and 10–50%)			10–50% vs (0–1% and 1–5% and 5–10%)			Test of all slopes are 0
Explanatory variable	Dependent	Coefficient	Odds ratio	*p* value	Coefficient	Odds ratio	*p* value	Coefficient	Odds ratio	*p* value	*p* value
Share of timber in the municipal income (%)	Ownership objective Timber sales^a^	− **0.96280**	**2.62**	**0.02**	− **1.52320**	**4.59**	**0.01**	− **2.71340**	**15.08**	**0.00**	**0.00**
	Ownership objective Fuelwood provision^a^	− **0.78850**	**2.20**	**0.05**	− **0.69430**	**2.00**	**0.17**	− **1.75030**	**5.76**	**0.01**	**0.02**
	Ownership objective Water resources^a^	− 0.54810	1.73	0.17	0.17980	0.84	0.73	− 0.02360	1.02	0.97	0.45
	Ownership objective Game and hunting^a^	− 0.27350	1.31	0.48	− 0.31750	1.37	0.52	0.01390	0.99	0.98	0.86
	Ownership objective Recreation^a^	0.13780	0.87	0.73	0.15400	0.86	0.76	1.08020	0.34	0.08	0.39
	Ownership objective Continued tradition^a^	− **1.42350**	**4.15**	**0.00**	− **1.36190**	**3.90**	**0.02**	− **1.04860**	**2.85**	**0.12**	**0.00**
	Ownership objective Non-wood forest products^a^	− 0.44020	1.55	0.28	− 0.41840	1.52	0.43	0.99560	0.37	0.12	0.15
	Index of economic objectives^a^	− **1.04193**	**2.83**	**0.00**	− **1.26337**	**3.54**	**0.01**	− **2.37476**	**10.75**	**0.00**	**0.00**
	Index of environmental objectives^a^	− 0.18279	1.20	0.64	− 0.02018	1.22	0.67	0.65816	0.52	0.24	0.56
	Index of recreation objectives^a^	− 0.05207	1.05	0.89	0.16088	0.85	0.73	1.22202	0.29	0.03	0.13
	Willingness to forego income for environmental priorities	0.50590	0.66	0.16	0.82920	2.29	0.08	0.50140	1.65	0.37	0.26
	Willingness to forego income for recreation	0.21680	1.24	0.56	0.39200	1.48	0.42	0.60150	1.82	0.30	0.68

Results statistically significant at 0.10 significance level are in bold

^a^The order of responses is inverted

There is a weak positive relationship between municipalities’ relative forest cover and timber sales as an ownership objective (odds ratio = 0.98, *p* = 0.09; Table 4) as well as a weak negative relationship between municipalities’ relative forest cover and the index of willingness to forgo timber income for environmental priorities (odds ratio = 1.01, *p* = 0.08; Table 4). However, forest cover is not significantly related to any measure of recreational priorities (Fig. 4b and Table 4). The position of the responding person did not significantly influence any of the relationships between the explanatory variables and the objectives of forest ownership.

**Table 4:** Summary of ordinal logistic regression results for relationships between ownership objectives and characteristics of municipalities

Explanatory variable	Dependent	Coefficient	Odds ratio	*p* value
Forest cover (%)	Ownership objective timber sales^a^	− **0.01497**	**0.98**	**0.09**
	Ownership objective fuelwood provision^a^	− 0.00440	1.00	0.61
	Ownership objective water resources^a^	**0.01920**	**1.02**	**0.03**
	Ownership objective game and hunting^a^	**0.02420**	**1.02**	**0.01**
	Ownership objective recreation^a^	0.00980	1.01	0.27
	Ownership objective continued tradition^a^	0.00150	1.00	0.87
	Ownership objective non-wood forest products^a^	− 0.00240	1.00	0.79
	Index of economic objectives^a^	− 0.01017	0.99	0.22
	Index of environmental objectives^a^	0.01226	1.01	0.13
	Index of recreation objectives^a^	0.00670	1.01	0.41
	Willingness to forego income for environmental priorities	**0.01440**	**1.01**	**0.08**
	Willingness to forego income for recreation	0.00800	1.01	0.34
Population (cap.)	Ownership objective timber sales^a^	− 0.00003	1.00	0.13
	Ownership objective fuelwood provision^a^	0.00001	1.00	0.11
	Ownership objective water resources^a^	− 0.00001	1.00	0.33
	Ownership objective game and hunting^a^	− 0.00001	1.00	0.43
	Ownership objective recreation^a^	− 0.00002	1.00	0.33
	Ownership objective continued tradition^a^	0.00001	1.00	0.22
	Ownership objective non-wood forest products^a^	0.00000	1.00	0.46
	Index of economic objectives^a^	**0.00001**	**1.00**	**0.03**
	Index of environmental objectives^a^	− **0.00002**	**1.00**	**0.07**
	Index of recreation objectives^a^	− 0.00001	1.00	0.16
	Willingness to forego income for environmental priorities	− 0.00001	1.00	0.17
	Willingness to forego income for recreation	− 0.00002	1.00	0.19
Population trend 2001–2021 (%)	Ownership objective timber sales^a^	0.00162	1.00	0.80
	Ownership objective fuelwood provision^a^	− 0.00628	0.99	0.35
	Ownership objective water resources^a^	0.00532	1.01	0.42
	Ownership objective game and hunting^a^	− 0.00105	1.00	0.87
	Ownership objective recreation^a^	− 0.00405	1.00	0.55
	Ownership objective continued tradition^a^	0.00439	1.00	0.52
	Ownership objective on-wood forest products^a^	− 0.00167	1.00	0.80
	Index of economic objectives^a^	− 0.00050	1.00	0.61
	Index of environmental objectives^a^	**0.01538**	**1.02**	**0.01**
	Index of recreation objectives^a^	0.00658	1.01	0.28
	Willingness to forego income for environmental priorities	− 0.00578	0.99	0.34
	Willingness to forego income for recreation	-0.00046	1.00	0.94
Share of rural sectors in local businesses	Ownership objective timber sales^a^	− **0.07722**	**0.92**	**0.00**
	Ownership objective fuelwood provision^a^	− **0.04436**	**0.95**	**0.04**
	Ownership objective water resources^a^	0.01140	1.01	0.60
	Ownership objective game and hunting^a^	− 0.02953	0.97	0.17
	Ownership objective recreation^a^	**0.04157**	**1.04**	**0.06**
	Ownership objective continued tradition^a^	− **0.06246**	**0.94**	**0.02**
	Ownership objective non-wood forest products^a^	− 0.02849	0.97	0.20
	Index of economic objectives^a^	− **0.06269**	**0.94**	**0.00**
	Index of environmental objectives^a^	0.01802	1.02	0.36
	Index of recreation objectives^a^	**0.05122**	**1.05**	**0.01**
	Willingness to forego income for environmental priorities	0.02908	1.03	0.14
	Willingness to forego income for recreation	**0.03869**	**1.04**	**0.06**

Results statistically significant at 0.10 significance level are in bold

^a^The order of responses is inverted

### Variation by municipal character

Municipal preferences are only weakly influenced by the character of the municipality. The population size is not significantly linked to the preferences of the municipality, apart from very weak negative relationship with the index of economic ownership objectives (odds ratio = 1.00, *p* = 0.03; Table 4) and weak positive relationship with the index of environmental ownership objectives (odds ratio = 1.00, *p* = 0.07; Table 4). The share of rural sectors in local businesses is positively associated with timber sales (odds ratio = 0.92, *p* < 0.01; Table 4), fuelwood provision (odds ratio = 0.95, *p* = 0.04; Table 4 and Fig. S1) and continued tradition of forest ownership (odds ratio = 0.94, *p* = 0.02; Fig. 4d and Table 4) as objectives of municipal forest ownership. The same indicator of rural nature of municipality is negatively related with several measures of recreational priorities (Table 4 and Fig. S1).

## Discussion

### Municipal priorities

Municipalities prioritise some non-market objectives over income generation (Fig. 2). In particular, recreational uses (including informal gathering of non-wood forest products) and water resources are highly ranked. Even the sale of inexpensive fuelwood to local households—a common practice of Czech forest owners—is considered about as important as income from commercial timber marketing. Municipalities also indicate that their management strategies are guided by environmental rather than economic goals (Table 2). While a small minority of local governments strongly prefer timber production over environmental and recreational objectives, most municipal owners appear to aspire to find synergies between market and non-market forest benefits.

Seen from the perspective of urban forestry, which tends to prioritise recreational uses (Konijnendijk et al. 2006), these results would not be surprising. However, urban forestry is hardly directly relevant for many of the approximately 200 000 km^2^ of municipal forests in Europe. Trivially, given a median population of just over 1000 in our sample, the Czech forest owning municipalities are mostly rural. Furthermore, property rights do not necessarily imply spatial proximity. Municipal properties may be located far from the municipality. To use an extreme example, several Czech towns own or have until recently owned forest land in Germany.

These findings suggest that non-market functions are also a major priority for many municipalities outside of urban contexts. They appear to be consistent with the presumed focus of public ownership and align with a broader shift in European municipal forestry towards multifunctional objectives (FAO and UNECE 2019). Elsewhere in Europe, researchers have repeatedly found that Nordic local governments emphasise multiple functions of their forest assets, or explicitly prefer non-market functions (Mattila et al. 2013; Nielsen et al. 2013; Richnau et al. 2013).

Timber sales and non-market uses are not necessarily contradictory. Europeans generally tend to prefer mixed forests, relatively open, with many old trees and few clearcuts (Edwards et al. 2012; Eriksson et al. 2015; Ciesielski and Stereńczak 2018), i.e. they value attributes that would preclude at least some of the intensive production models. However, landowners often provide for recreational use through measures that are consistent with commercial timber output, such as dense recreational facilities.

Nevertheless, other findings indicate that municipalities’ perspective may be more complex than a simple dilemma between timber sales and non-market uses. Continued tradition of forest ownership is an even higher-ranking objective than recreation and water resources. Also, continuation of a pre-existing practice is the third most common goal of current forest management practice. These results suggest that for some municipalities the primary motivation for owning forest is the simple fact of pre-existing historical ownership, rather than any particular priority, and that they manage them—with an eye to technological change and within the constraints of evolving regulations—in the way they are accustomed to.

Municipal forests in Europe “often showed a long ownership and management continuity that was aimed at the enduring welfare of the community” (Mölder et al. 2021: 200). After the conclusion of the restitution process around 2005, there was no significant disinvestment from Czech municipal forests—even during the timber market crisis after 2017 (Hlásny et al. 2021), the area of municipal forest property decreased by only 0.11%. The passive embrace of an ownership tradition could be conceptualised as institutional inertia. However, municipal decision-makers may view it as an exercise of extended responsibility for the inheritance.

Interestingly, there seems to be a similar perception of sharing over time among private forest owners (e.g. Kline et al. 2000; Hugosson and Ingemarson 2004; Richnau et al. 2013; Feliciano et al. 2017; Koskela and Karppinen 2021; Tiebel et al. 2021). Among others, Feliciano et al. (2017) found a strong sense of intergenerational stewardship among private forest owners in Europe. Although their respondents emphasised the connection with future owners rather than inheritance from ancestors, these concepts are likely to be similar and perhaps overlapping. The professional culture of European forestry has traditionally accentuated long-term planning and recognition of distant time horizons spanning multiple generations of owners and managers (Glück 1987; Hoogstra and Schanz 2009).

The more or less passive assumption of the pre-existing ownership tradition could be a complicating factor in the transition to multifunctional approaches and, in particular, the practical integration of non-market services. Forest ownership based on continuity may inadvertently tend to perpetuate existing silvicultural practices. These practices are likely to focus solely on traditional raw material production. We speculate that, while municipalities often have a vague preference for recreational and environmental objectives in principle, their forest management models may remain geared towards timber output and little else.

Such a mismatch between the relative prioritisation of environmental or recreational objectives and day-to-day forestry practices could be exacerbated by several contributing factors. Mattila et al. (2013) concluded that multifunctional management, although preferred by municipalities, may be difficult to implement. Owners lack clear success criteria for non-market benefits, analogous to net income in productive forests; also, governance of various forest functions is fragmented across local government departments, and current staff experience is in traditional intensive forestry (Mattila et al. 2013). Furthermore, while the concept of multifunctional forestry itself provides a useful shared ideology for various interests, it remains “abstract and vague…with highly diverse conceptual and managerial interpretations” (Hoogstra-Klein et al. 2017: 256). Another complicating factor is the formal allocation by the government of one of the three spatially explicit “forest functions” (productive, protective or social/special purpose) to individual forest tracts in some Central European countries (Bončina et al. 2019): while municipal decision-makers have stated their objectives when prompted, in practice they may ultimately tend to assume that prioritisation is the government regulator’s responsibility.

### Variation of priorities

There is some, albeit mostly small, variation in municipal priorities. Not surprisingly, local governments with a higher share of timber sales in their municipal income are more likely to favour traditional economic objectives (Table 3 and Fig. 4). Similarly, the preference of private forest owners for nature conservation is negatively related to the proportion of their income derived from forestry (Ingemarson et al. 2006). Municipalities with more rural character also seem to place greater emphasis on income generation (Table 4). We interpret that timber income remains relatively more important for smaller, peripheral communities with resource-based economies.

While larger and less rural municipalities—again predictably—are generally more likely to emphasise environmental priorities, this link seems to be less systematic (Table 4). In particular, the lack of any relationship between either population size or local forest cover and recreational objectives (Table 4 and Fig. 4) suggests that the relative need for and supply (or deficit) of recreational resources is not a major driver for local governments to prioritise them in their forest planning.

A combination of several factors may be at work in this pattern. Rural and urban publics in Europe seem to share broadly similar forest preferences (Ciesielski and Stereńczak 2018), so their ideological priorities may ultimately overlap as well. Moreover, municipal decision-makers may be less able to explicitly conceptualise intangible non-market priorities and reflect on their materiality than is the case with timber sales, a measurable outcome. Finally, the need to consider values in prioritisation is less pressing for non-rivalrous goods such as recreational opportunities, as local governments can assume that these could be well provided on someone else’s land. Ownership is not necessarily a critical prerequisite of non-market uses in many European countries with freedom to roam laws. As Bouriard and Schmithüsen (2005) pointed out, forest ownership is often seen as ownership of timber.

Nevertheless, the relatively low variance in preferences among Czech municipalities is probably the most relevant finding (Tables 3 and 4). Typically, local governments appear to strive for an emphasis on multiple functions of their forest. Current day-to-day silvicultural operations may remain focused on traditional timber production, but stated objectives could suggest a broader ambition.

Therefore, and somewhat unexpectedly, urban forestry (in the narrow sense of woodlands per se) in Europe could provide a useful guidance for broader municipal practice. Urban silviculture often involves a mixture of recreational and timber production objectives. A better understanding of its practical experience with synergies between the two functions, and how specifically this experience could be used to support the needs of municipal forestry outside urban contexts, are potentially important directions for further research.

## Conclusions

Municipalities are important forest owners in some European countries, including rural local governments and land distant from built-up areas. This study suggests that Czech municipal owners seek synergies between market and non-market forest uses, and that most of them prioritise recreational and environmental objectives over income generation. Despite some heterogeneity, these preferences are surprisingly similar in urban and rural areas. This is particularly the case for recreational resources, where municipal planning may be driven by value preferences rather than potential material needs.

These results may potentially have important policy implications. Ultimately, the priorities of wider municipal forestry appear to align with those of urban forestry. This suggests a way forward for multifunctional forestry approaches, as well as wider involvement of municipal forests in conservation and recreation objectives beyond urban and suburban spaces. However, practical constraints and municipalities’ emphasis on continuity may reinforce path dependencies in silvicultural practice.

## Supplementary Information

Below is the link to the electronic supplementary material.

Supplementary file1 (PDF 666 kb)

## Data Availability

The original research data are available on 10.5281/zenodo.15121993.
