# Supplementary material for: Municipal governments’ perspectives on forest ownership: Insights from Czechia
Source: Ambio. 2025 Aug 9;55(2):402–14. doi: 10.1007/s13280-025-02231-8 (PMC12779864; doi:10.1007/s13280-025-02231-8)
Supplement: Supplementary file 1 — Supplementary file1 (PDF 666 kb) [file 13280_2025_2231_MOESM1_ESM.pdf]

Ambio

Supplementary Information
This supplementary information has not been peer reviewed.

Title: **Municipal governments' perspectives on forest ownership: Insights from Czechia**

Figure S1. Ordinal logistic regression results for the relationships between (a) share of rural sectors in local businesses (%) and ownership objective Timber sales, (b) share of rural sectors in local businesses (%) and ownership objective Fuelwood provision, (c) share of rural sectors in local businesses (%) and ownership objective Recreation, (d) forest cover (%) and ownership objective Timber sales, (e) forest cover (%) and ownership objective Water resources, (f) forest cover (%) and ownership objective Game and hunting, (g) share of timber sales in the municipal income (%) and ownership objective Continued tradition of forest ownership. All significant relationships that are not shown in Fig. 4 are reported here. Details of the ownership objective variables are given in Table 1.

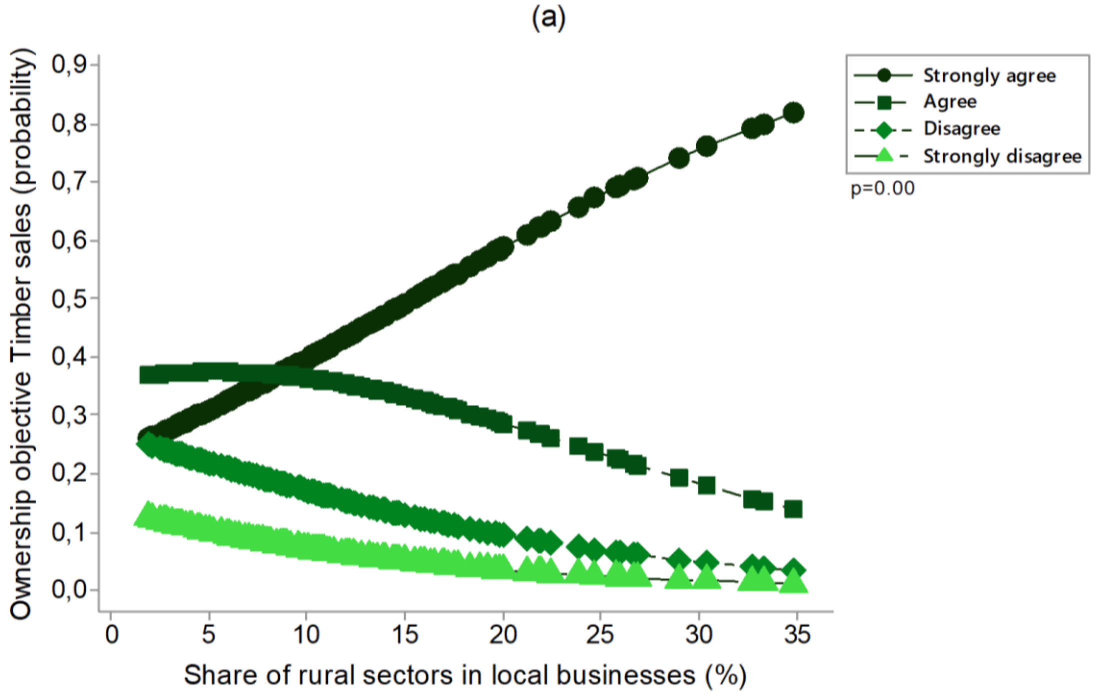

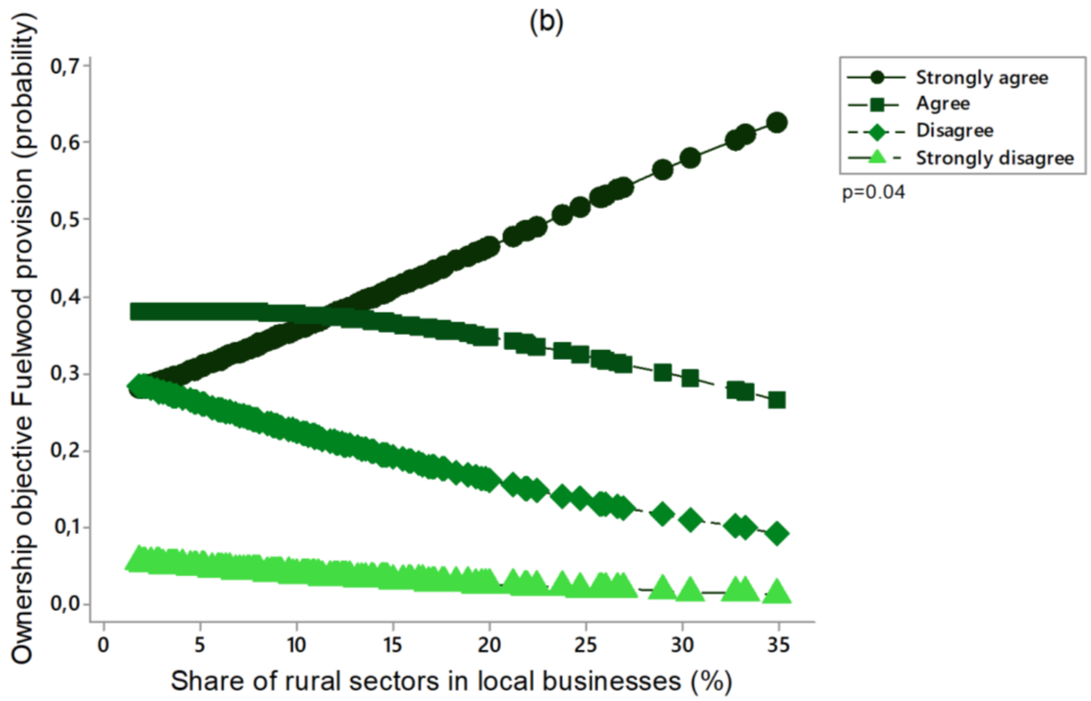
(b)
Ownership objective Fuelwood provision (probability)
0,7
0,6
0,5
0,4
0,3
0,2
0,1
0,0
0
5
10
15
20
25
30
35
Share of rural sectors in local businesses (%)
Strongly agree
Agree
Disagree
Strongly disagree
p=0.04

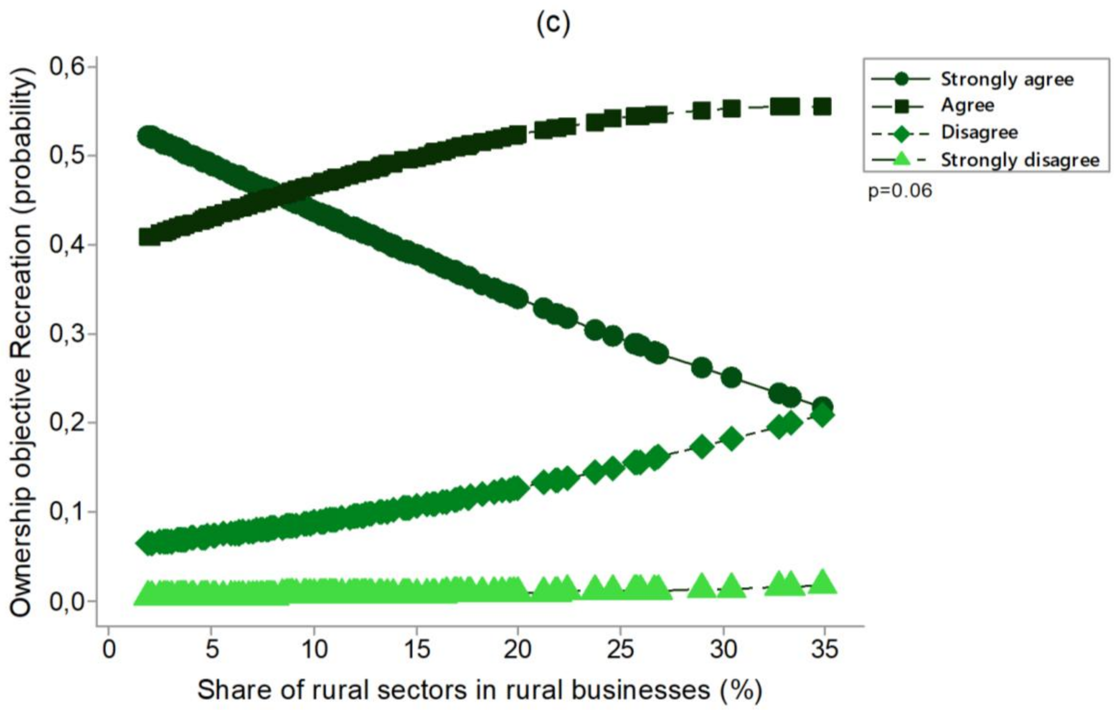
(c)
Ownership objective Recreation (probability)
0,6
0,5
0,4
0,3
0,2
0,1
0,0
0
5
10
15
20
25
30
35
Share of rural sectors in rural businesses (%)
Strongly agree
Agree
Disagree
Strongly disagree
p=0.06

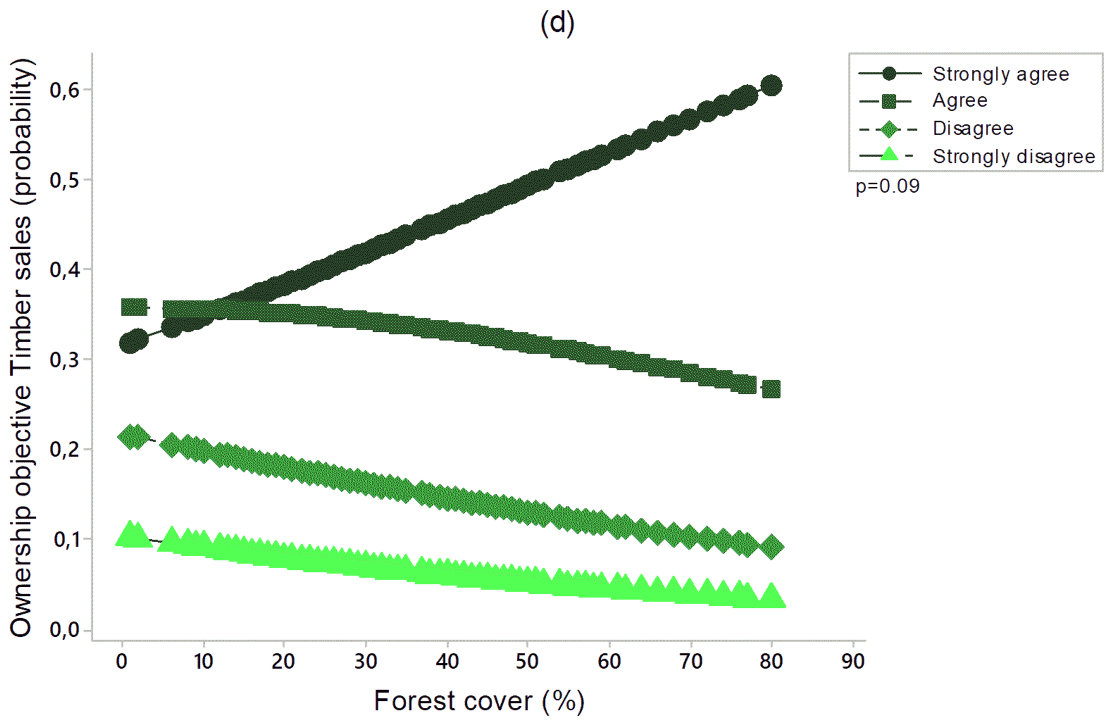
(d)
Ownership objective Timber sales (probability)
0,6
0,5
0,4
0,3
0,2
0,1
0,0
0
10
20
30
40
50
60
70
80
90
Forest cover (%)
Strongly agree
Agree
Disagree
Strongly disagree
p=0.09

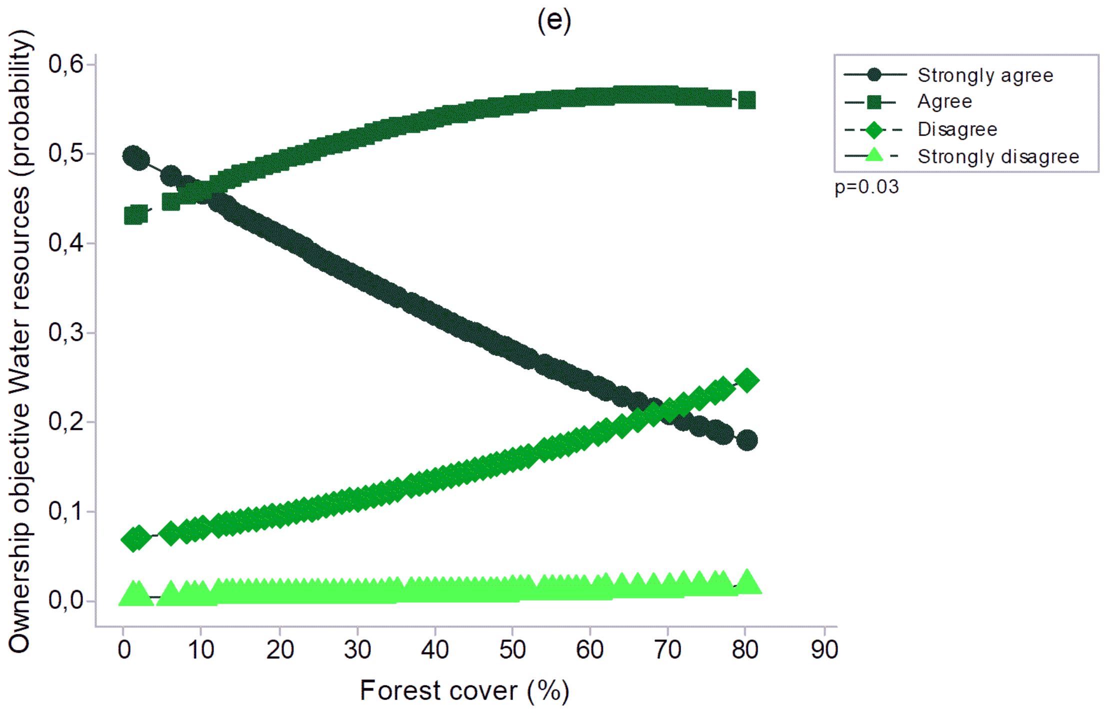
(e)
Ownership objective Water resources (probability)
0,6
0,5
0,4
0,3
0,2
0,1
0,0
0
10
20
30
40
50
60
70
80
90
Forest cover (%)
Strongly agree
Agree
Disagree
Strongly disagree
p=0.03

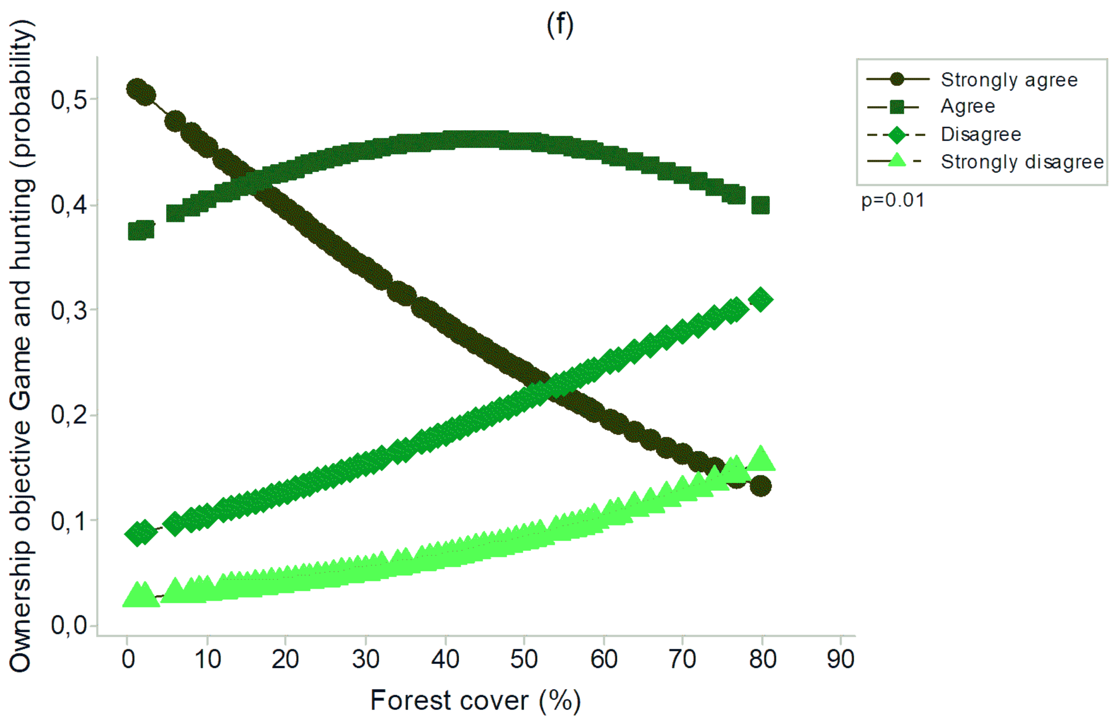
(f)
Ownership objective Game and hunting (probability)
0,0
0,1
0,2
0,3
0,4
0,5
0
10
20
30
40
50
60
70
80
90
Forest cover (%)
Strongly agree
Agree
Disagree
Strongly disagree
p=0.01

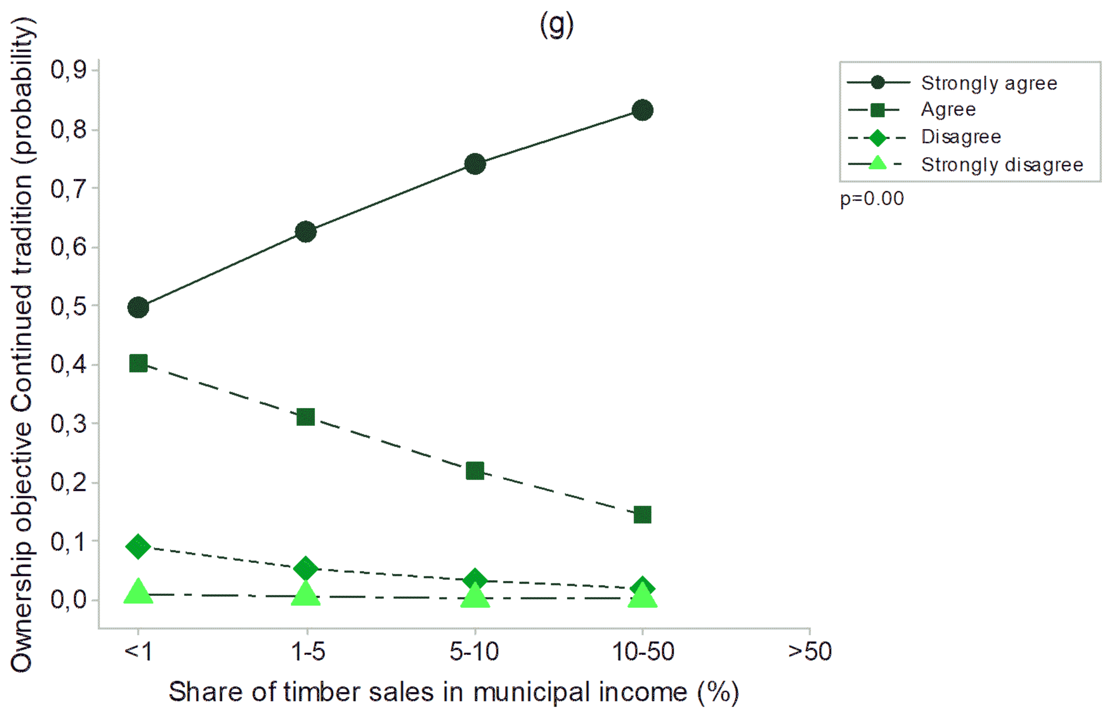
(g)
Ownership objective Continued tradition (probability)
0,0
0,1
0,2
0,3
0,4
0,5
0,6
0,7
0,8
0,9
<1
1-5
5-10
10-50
>50
Share of timber sales in municipal income (%)
Strongly agree
Agree
Disagree
Strongly disagree
p=0.00
